# Supplementary material for: Comparative multiomics analysis of cell physiological state after culture in a basket bioreactor
Source: Sci Rep. 2022 Nov 23;12:20161. doi: 10.1038/s41598-022-24687-4 (PMC9686226; doi:10.1038/s41598-022-24687-4)
Supplement: Supplementary file 1 — Supplementary Information 1. [file 41598_2022_24687_MOESM1_ESM.zip › raw data/Transcriptomic raw data/7.AS/2.dasplot/TE1vsTC1/SE/19_DVL3_NW_023666063.1_14040132_14040267_-@NW_023666063.1_14039861_14039954_-@NW_023666063.1_14039576_14039645_-.pdf]

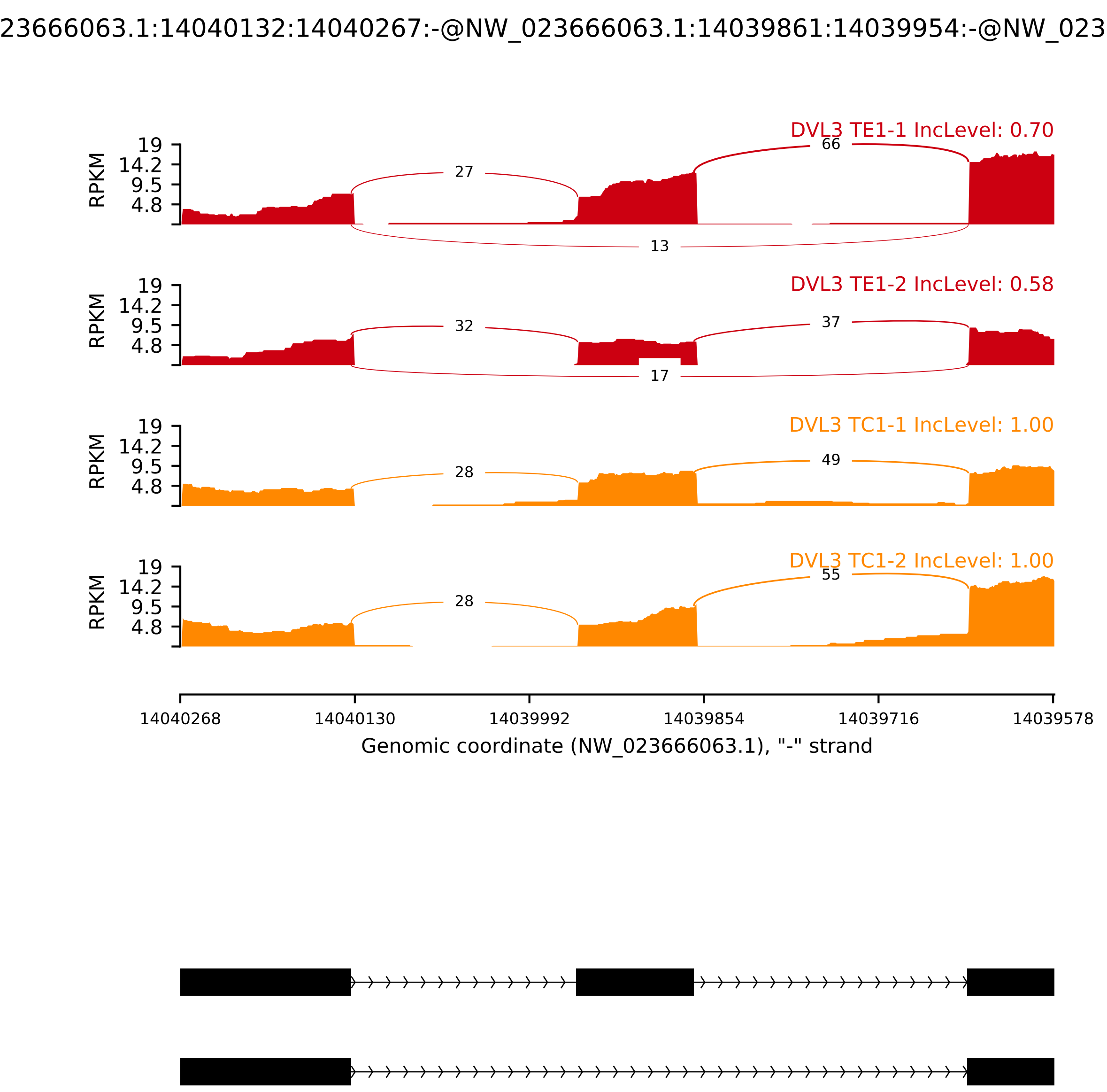
23666063.1:14040132:14040267:-@NW_023666063.1:14039861:14039954:-@NW_023666063.1:14039576:1403
DVL3 TE1-1 IncLevel: 0.70
DVL3 TE1-2 IncLevel: 0.58
DVL3 TC1-1 IncLevel: 1.00
DVL3 TC1-2 IncLevel: 1.00
RPKM
19
14.2
9.5
4.8
27
66
13
32
37
17
28
49
28
55
14040268
14040130
14039992
14039854
14039716
14039578
Genomic coordinate (NW_023666063.1), "-" strand
